# Design of a study evaluating the effects, health economics, and stakeholder perspectives of a multi-component occupational rehabilitation program with an added workplace intervention - a  study protocol

**DOI:** 10.1186/s12889-018-5130-5

**Published:** 2018-02-05

**Authors:** Marit B. Rise, Martin Skagseth, Nina E. Klevanger, Lene Aasdahl, Petter Borchgrevink, Chris Jensen, Hanne Tenggren, Vidar Halsteinli, Trym N. Jacobsen, Svein B. Løland, Roar Johnsen, Marius S. Fimland

**Affiliations:** 1 Department of Public Health and Nursing, Norwegian University of Science and Technology, Trondheim, Norway; 2 Department of Mental Health, Norwegian University of Science and Technology, 7491 Trondheim, Norway; 3 Department of Physical Medicine and Rehabilitation, St. Olav’s Hospital, Trondheim University Hospital, Trondheim, Norway; 4 Hysnes Rehabilitation Center, Trondheim University Hospital, Trondheim, Norway; 5 Department of Circulation and Medical Imaging, Faculty of Medicine, Norwegian University of Science and Technology, Trondheim, Norway; 6 National Centre for Occupational Rehabilitation, Rauland, Norway

**Keywords:** Absenteeism, Occupational health, Mental health, Return to work, Acceptance and commitment therapy, Cognitive behaviour therapy, Musculoskeletal diseases

## Abstract

**Background:**

Recent research has suggested that interventions at the workplace might be the most potent ingredient in return to work interventions, but few studies have investigated the different effects of workplace interventions as part of occupational rehabilitation programs. The comprehensive design described in this article includes effect (on return to work and health outcomes), and health economic evaluations of a workplace intervention added to a multicomponent rehabilitation program. Qualitative and mixed method studies will investigate sick-listed persons’, rehabilitation therapists’ and employers’ perspectives on the usability and outcomes of the rehabilitation program and the workplace intervention. The program and intervention are provided to patients with musculoskeletal, psychological or general and unspecified diagnoses. The program is multi-component and includes Acceptance and Commitment Therapy, physical exercise, patient education and creating a plan for increased work participation.

**Methods:**

Persons who are employed, aged from 18 to 60 years, with a current sick leave status of 50% or more and a diagnosis within the musculoskeletal, psychological or general and unspecified chapters of International Classification of Primary Care-2 (ICPC-2) will be recruited to a researcher-blinded parallel-group randomized controlled trial. All participants take part in an in-patient occupational rehabilitation program, while the intervention group also takes part in an intervention at the workplace. The effect and economic evaluation will investigate the effect of the added workplace intervention. The primary outcome measures will be time until full sustainable return to work and total number of sickness absence days in the 12 months after inclusion. Health economic evaluations will investigate the cost-effectiveness and cost-utility. Qualitative studies will investigate rehabilitation therapists’ experiences with working towards return to work within an ACT-approach and stakeholders’ experiences with the workplace intervention. A mixed methods study will combine quantitative and qualitative findings on the participants’ expectations and motivation for return to work.

**Discussion:**

The outline of this comprehensive study could represent an important addition to the standard designs of return to work evaluation. The mixed methods design, with qualitative approaches as well as a rigorous randomized controlled trial, might prove useful to shed light on contextual factors.

**Trial registration:**

ClinicalTrials.gov: NCT02541890. September 4, 2015.

## Background

Work disability is considered one of the biggest challenges for governments and policy makers in The organisation for economic co-operation and development (OECD) countries [1]. In Norway, sickness absence rates are the highest in the OECD area [2], with costs of disability benefits totaling to approximately 4–5% of GDP in 2010 [1]. Hence, many European countries, including Norway, have implemented legislation and acts to prevent long-term absence and work disability due to health problems [3], and many rehabilitation programs have been established to help patients return to work (RTW) [3–9]. In Norway, inpatient occupational rehabilitation programs are widespread. They usually consist of group-based and individual cognitive behavioral- and physical exercise sessions, as well as patient education [10–12], but have rarely included workplace interventions [13]. Individuals are usually referred to such programs by their general practitioner, but can also be referred from the Welfare and Labor Service.

So far, most occupational rehabilitation programs have been tailored for specific diagnosis groups, most often for musculoskeletal disorders [7, 14–17] and some for mental health problems [18–21]. Several of these programs have shown effect on return to work and other outcomes [7, 14]. Different models have been used to help understand work disability; the medical model, the biopsychosocial model, and the case management ecological model, where the environmental influence has gradually become more important. Now, political, economic, cultural and work place environment are considered to interact with the persons own behavior and attitudes [22]. Thus, sickness absence is no longer solely attributed to the person’s health problems, but is considered a result of complex interactions between the person’s physical and behavioral characteristics and the socio-cultural environment, including the health services, the work environment and the system providing financial compensation [22, 23]. Together with an increased focus on the high level of co-morbidity among sick-listed persons [24–27], these developments warrant more generic programs in order to be useful for patients with different and overlapping diagnoses. The effect of generic programs are still unclear. In addition, many have argued that interventions to prevent work disability and long-term sickness-absence should address all stakeholders and systems involved in this field [28, 29]. Employers have a key role in the return to work process [1] that seems to be confirmed in systematic literature reviews [30–32]. Similarly, the need for collaboration between different types of health care professionals in managing and preventing disability has also been highlighted in the literature [16, 22, 33]. Collaboration and coordination between the different actors is thus needed to prevent prolonged sickness absence and to improve return to work [22, 34].

Workplace interventions are defined as initiatives linked to the workplace, including adaptations at work or involvement of the work-environment stakeholders. A landmark study from Canada suggested that interventions at the workplace might be the most potent ingredient in occupational rehabilitation, especially when combined with a clinical intervention [16]. The study was later partly replicated in the Netherlands and Denmark [7, 35, 36]. In a Dutch study Lambeek et al. [7] found that a workplace intervention as part of a clinical rehabilitation program was more effective in facilitating return to work than usual care. However, the effects of specific intervention components are uncertain and publication bias is present [37]. Several systematic reviews have reported that, for patients with back pain, and to some extent for patients with mental disorders, multimodal rehabilitation which includes interventions at the workplace is more effective than other interventions [30–32, 38]. However, the effect of workplace interventions on sustainable return to work is so far unclear [32]. It seems important to involve the different actors, such as patient and employer, in active consultation and consensus about how to modify the work environment, specific work tasks or the work organization to facilitate return to work [38, 39], and this collaborative approach is recommended [29].

Hence, current knowledge points towards more multimodal and generic occupational rehabilitation programs that comprise the different stakeholders in the return to work process, including the workplace. There is thus a need for effect studies which can attend to the complexity of multimodal interventions, as well as more heterogeneous patient groups. In addition to randomized controlled designs to investigate effects on return to work, qualitative methodological approaches should focus on the experiences of the different stakeholders in the return to work process (sick listed persons, service providers, and employers), and help uncover any unknown facilitators and barriers in the return to work process. Qualitative methodological approaches could also investigate the stakeholders’ perceptions of usability and other potential outcomes from initiatives to support work participation and return to work. It is also necessary to assess the cost-benefit of such interventions through economic evaluations.

## Objectives

**Fig. 1:**
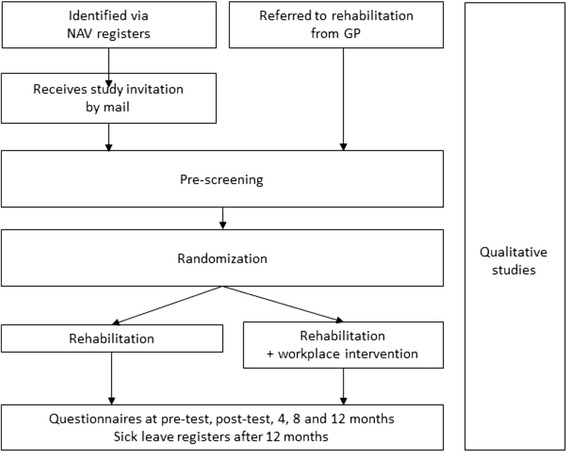
Study design

The objective of this article is to describe the protocol of a study which endeavors to encompass all the requirements described above. The proposed study design includes an effect evaluation (a randomized controlled trial), an economic evaluation, two qualitative studies and one mixed methods study which combines qualitative and quantitative data. The proposed study design is presented in Fig. 1.

The development of this study and the study design is based on the experiences from a large randomized controlled trial (RCT) conducted in Central Norway [11, 12, 40]. Interviews with rehabilitation therapists and participants made it clear that very few participants had been in contact with the workplace or employer during the rehabilitation program. A qualitative study also showed that the participants, at the end of the program, perceived return to work as a long and complex process, and few of them felt ready to return to work [41]. To enhance the return to work focus and improve the return to work rates, the researchers, in collaboration with the clinic, developed and piloted an intervention at the participants’ workplace. This, added to the rehabilitation program, is proposed as the intervention in the RCT.

Based on the findings of high comorbidity in sick-listed persons, and to encompass the largest group of sick-listed persons in Norway, the rehabilitation programs and the workplace intervention are developed specifically for a heterogeneous patient group; patients with a diagnosis within the musculoskeletal, psychological or general and unspecified chapters of International Classification of Primary Care-2 (ICPC-2). Large part of the program’s psychological therapy is provided in groups where persons with different diagnoses are mixed. This is still a rather new approach in occupational rehabilitation. The therapists' educational backgrounds are also heterogeneous, including backgrounds such as nurses, physiotherapists, psychologists, and social workers. All were provided training and supervision in the same psychological therapy approach. Acceptance and Commitment Therapy (ACT) [42, 43] is chosen as the psychological therapy approach. The ACT approach has, so far, very limited use in occupational therapy [40, 44, 45].

### The Norwegian sickness absence system

All legal residents in Norway are included in the Norwegian public insurance system. Medically certified sick leave is compensated with 100% coverage for the first 12 months. The medical certificate can, and is encouraged if possible, to be graded from 20% to 100%, independent of employment fraction. This means that if you are employed in 50% position and are 50% on sick leave, you are working 25%. After 12 months of sick leave, it is possible to apply for long-term medical benefits, compensating approximately 66% of the income.

## Methods

The aims of the studies are to:

1. Through a randomized controlled trial, investigate whether an occupational rehabilitation program with a workplace intervention can improve sustainable return to work and reduce sickness absence, compared to an occupational rehabilitation program without a workplace intervention.
2. Through an economic evaluation, assess whether an occupational rehabilitation program with a workplace intervention is cost effective, compared to a program without a workplace intervention.
3. Through two qualitative studies and one mixed methods study, investigate the patients, rehabilitation therapists, and employers’ experiences of the usability and perceived outcomes of the programs and the intervention.

### Participants and recruitment

People living in Trøndelag (two counties in Central Norway) are eligible for inclusion if they 1) are aged 18 to 60 years, 2) are sick-listed 2–12 months, 3) hold at least a 20% job position (e.g. minimum one day per week), 4) have an employer, 5) have a sick-leave status of at least 50% off work, 6) anticipate at least four more weeks of sick-leave, and 7) have a diagnosis within the musculoskeletal, psychological or general and unspecified chapters of ICPC-2. The participants’ diagnoses are as assessed by the participants’ general practitioner (GP). In addition, potential participants must currently have an employer. Exclusion criteria are: Being self-employed, having or being under consideration for a serious somatic or mental health/substance abuse disorder (assessed by the screening clinic), currently undergoing rehabilitation, having significant problems with working in a group (assessed by the screening clinic based on questionnaires and clinical interview), insufficient comprehension of Norwegian language to participate in group sessions and to fill out questionnaires, scheduled for surgery within the next 6 months, or being pregnant.

#### Sample size

Sample size is calculated in line with the requirements in the randomized controlled trial. Comparison of return to work with Kaplan Meier survival analysis with log rank test with a hazard ratio of 0.6 (alpha 0.05, beta 0.20) would require 63 participants in each group. As we use register-based sickness absence data, there will be no loss to follow-up in the intention-to-treat analyses of primary outcomes. However, to provide greater statistical power for questionnaire-based outcomes we will include at least 80 participants in each group. The sample size estimation was based on results from previous studies in this field [7, 16, 36], as well as preliminary results from ongoing studies [11].

#### Recruitment to the effect evaluation and economic evaluation

Potential participants are recruited in two ways. 1) The Norwegian Labour and Welfare Service (NAV) will provide monthly lists of persons meeting the inclusion criteria. An assigned project co-worker sends out invitations to potential participants who accept to participate by contacting the research group. Persons who accept to participate are invited to an out-patient screening clinic for clinical examination and interview before inclusion. 2) General practitioners (GPs) refer patients to the study and the rehabilitation program. The GPs will be provided with information of the project through written leaflets, the website of the rehabilitation centre (www.stolav.no/hysneshelsefort), and can call a project co-worker for any questions. The GPs will refer potential participants directly to the out-patient screening clinic for clinical examination and interview. Besides the project co-worker who sends out the invitation letters, none of the researchers in the project will know the identity of potential participant, until the person accepts to participate through answering the invitation letter or accepts to being referred to the program by their GP.

All participants will complete baseline study outcome measurements and several screening questionnaires before an out-patient screening is performed. The screening is conducted by a medical doctor, a psychologist and physiotherapist during clinical examination and interview. The clinic assesses the final eligibility before randomization and excludes all persons who do not fit the inclusion criteria. The personnel at the screening clinic will also give oral and written information about the study, and emphasize that agreeing to participate includes being randomized to a program with or without a workplace intervention. All participants who are included by the screening clinic will sign a written consent form prior to randomization. Taking part in the RCT will be a prerequisite for participation in the rehabilitation program and the intervention. The participants can decline participation and withdraw from the study (and program) at any point.

#### Randomization

Persons who are found eligible and sign an informed consent will be randomized to the intervention arm (rehabilitation program with a workplace intervention) or the control arm (rehabilitation program only). Block randomization with unknown block sizes is performed using a web-based program delivered by a third party - the Unit for Applied Clinical Research at the Norwegian University of Science and Technology.

#### Recruitment to qualitative and mixed methods studies

All participants in the RCT will be eligible to take part in qualitative studies. Participants will be recruited strategically to inform the specific research aims. Potential participants will be given written and oral information about the studies and all participants will sign a consent form before taking part in interviews or observation. Potential participants in the first of the two qualitative studies are all rehabilitation therapists at the center, including all those providing the workplace intervention. They will be contacted directly by the researchers, and we aim to recruit 10–15 therapists. For the observation in the second qualitative study, all participants in one program group will receive written information and asked to consent to having a researcher present in their group during the rehabilitation program. All participants in the group must consent to the observation. Potential participants in the second qualitative study are the participants in the intervention arm, their employers and rehabilitation therapists. First, the participant will be given written information, and asked to take part in interviews and whether a researcher can observe the workplace intervention. If the participant consents, their employer will be contacted with the same information. The rehabilitation therapist will also be asked by the researcher to take part in interviews. We aim to recruit a sample where the participants’ gender, type of occupation, age, and length of sickness absence is varied. We aim to recruit 10–15 triads (participant, therapist and employer). Potential participants in the mixed methods study are the participants (from intervention group and control group) who have completed questionnaires 4 months after the end of the rehabilitation program. A sample of approximately 20 of these participants will be asked to take part in individual interviews. This will be a strategic sample to explore their perspectives on the return to work process in interviews four months after the rehabilitation programs, and to elaborate on quantitative data already collected in the RCT on the same subject.

### The occupational rehabilitation program

Regardless of group allocation in the RCT, all study participants take part in an occupational rehabilitation program. The program is composed of elements that are assumed to be useful in occupational rehabilitation for long-term sickness, and includes physical exercise, psychological therapy, and work related problem solving. Hysnes Rehabilitation Center (www.stolav.no/hysneshelsefort) is the setting for the occupational rehabilitation programs. The center was established as part of St. Olav’s Hospital and is located in the municipality of Rissa, a 50 min boat ride or 90 min drive from Trondheim, the third largest city (181.500 inhabitants) in Norway. The center has provided in-patient occupational rehabilitation programs since 2010.

The rehabilitation program lasts four weeks; the first two weeks at the rehabilitation center, the third week at home, and the fourth week at the center, engaging participants in full ‘work’ days, aiming to establish normal daily routines. The program is provided by interdisciplinary teams consisting of psychologists, physiotherapists, exercise physiologists, nurses, physician and welfare case worker provide the programs. All professionals are trained as ACT therapists. Each therapist treats 2–4 clients individually and in groups, focusing on behavior change and coping. Two therapists are assigned to each group which includes 6–8 participants. A mid-way report and a final report are sent to the GP. If the participant agrees, the reports are also sent to the participant’s welfare office and employer.

#### Physical exercise

Physical exercise is utilized to improve health, fitness, functional capacities, and to reduce fear-avoidance related to physical activity. An exercise plan is made for each participant and follow-up is provided during the program, both in groups and individually. The main objective for individual follow-up of exercise during the program is to ensure that the exercise plan is suitable and realistic and can be useful for the participant after the program.

#### Acceptance and commitment therapy (ACT)

The psychological therapy approach is ACT, an approach which uses acceptance and mindfulness strategies, along with commitment and behaviour change strategies, to increase psychological flexibility [42, 43]. ACT aims to improve work functioning, general skills of psychological flexibility and motivation to return to work despite the presence of pain or other symptoms [43]. Psychological flexibility is described as “the ability to contact the present moment more fully as a conscious human being, and to change or persist in behaviour when doing so serves valued ends” [42, 43]. ACT is increasingly used in various settings and for different patient groups [46] and has shown positive results in several areas [47–51]. A written manual for the ACT sessions (both in groups and individually) has been made by health professionals at the rehabilitation centre in collaboration with an experienced psychologist. The manual describes the intended aims and therapeutic processes of each session, including content and techniques. For every session, it also gives examples of observable participant behaviour that could indicate an increase in psychological flexibility. To develop and strengthen the rehabilitations therapists’ use of the ACT approach, they receive continuous individual supervision from an experienced psychologist. In addition, visual recordings and observation of therapy sessions are used in individual and group supervision of the therapists, to strengthen fidelity to the approach.

#### Work-related problem solving

Work-related problem solving aims to identify challenges and possibilities regarding the return to work process, in order to create an individual return to work plan. Interaction with relevant stakeholders (such as employer, colleagues, GP, welfare case worker or family members), will be discussed, as well as commenced during the rehabilitation process if the participant finds it suitable and helpful. The participant thus chooses whether and how to involve any of these stakeholders. All participants will, in collaboration with their rehabilitation therapist, create their own return to work-plan with individually tailored elements. Here, relevant challenges, measures and solutions regarding the workplace and other parts of the participant’s life will be described (for example work adjustments, as well as goals for physical exercise and social life). The employer contributes to the part of the plan which concerns issues at the workplace, and receives this part of the final plan. If the participant consents, the employer receives the whole plan. In addition, the program includes educational sessions on sleep, pain, nutrition and work-related aspects. ACT is the common ground for and fuels every aspect of the program; an emphasis on developing generalizable and lasting behaviour change that the participants consciously choose in accordance with personal values.

### The workplace intervention

The workplace intervention is inspired by previous successful workplace interventions [7, 16, 52, 53], and was developed by professionals at the rehabilitation center and the research group, with input from the Norwegian labor and welfare administration. The intervention is adapted to the time frame of this rehabilitation program, as well as the geographical, sociocultural and socioeconomic context, also taking into account that these programs will be delivered within a specialist hospital system.

The workplace intervention will consist of a preparatory part during the first two weeks, a meeting at the workplace in the third week of the program, and follow-up work related to the meeting in week four. The individual therapy sessions during week 1 and 2 are used to map the participant’s work situation, as well as any potential challenges and possibilities in the return to work process. During the first two weeks of the rehabilitation program the employer is contacted and an appointment for the workplace meeting is made. A one-hour work-specific ACT group session is held during week 2. This session focuses on the participants’ perceptions related to the return to work process, and the possibilities of coping at work despite disabilities are explored.

The participant and the rehabilitation therapists will meet 15–30 min before the workplace meeting. If it is not agreed upon earlier, they will discuss any limitations of topics which will be discussed with the employer present. A tour on the workplace to view the work arrangements is conducted when found relevant by the participant and rehabilitation therapist. The meeting lasts approximately two hours, and includes the participant, his or her employer, and the participant’s rehabilitation therapist. From the employer, the participant’s closest leader should always attend, but other persons, such as employee representative or personnel safety representative, considered to be helpful for the return to work process (e.g. due to necessary decision making authority) could also take part. The participant’s GP and/or labor and welfare case worker will be informed about the meeting and will be involved when appropriate. The rehabilitation therapist chairs the meeting. The objective is to discuss and plan the participant’s return to work process and the actual return. This encompasses identifying the participant and employer’s views on any challenges in returning to work, and to discuss potential measures. Conclusions will be made and the responsibility for implementation of any measures will be established.

After the workplace meeting the rehabilitation therapist will contact the employer to ensure that actions agreed upon in the meeting are taken and that measures and solutions will be implemented. A report is sent to those who took part. The participant and the rehabilitation therapist subsequently will discuss the experiences and outcomes from the workplace meeting. The return to work plan is concluded and mailed to the participant’s general practitioner. The plan is mailed to the employer if the participant finds it helpful.

### Study design

The study includes three main parts; an effect evaluation, an economical evaluation, and two qualitative studies and one mixed methods study.

#### Effect and economic evaluation

The effect evaluation is conducted as a randomized controlled study with a parallel group design. There are three main research questions in the effect evaluation:

- Is an in-patient rehabilitation program with a workplace intervention more effective in increasing return to work (time until full sustainable return to work) and reducing sickness absence (total number of sickness absence days during 12 months of follow-up from inclusion), compared to the rehabilitation program only?
- Is an in-patient rehabilitation program with a workplace intervention more effective in improving health, quality of life, and readiness for return to work, compared with the rehabilitation program only?
- Is the in-patient rehabilitation program with a workplace intervention cost- effective, compared to the rehabilitation program only?

#### Qualitative and mixed methods studies

Three different studies will be conducted:

- A qualitative interview study will investigate how the rehabilitation therapists attend to the program’s main objective – return to work – within a rehabilitation program based on the ACT approach. This study will explore the rehabilitation therapists’ experiences with using a novel therapy approach to enhance return to work. (Study A)
- A qualitative interview and observation study will investigate the different stakeholders´ (sick-listed person, employer, and rehabilitation therapist’s) experiences of the feasibility and outcomes from a workplace intervention. (Study B)
- And a mixed methods study, including quantitative data from questionnaires and qualitative data from interviews, will investigate and elaborate on any changes in the participants’ expectations to return to work 4 months after a rehabilitation program with or without a workplace intervention. (Study C)

### Data collection

#### Effect evaluation

Data on sickness benefits and other social benefits will be based on register data from the National Welfare Administration. Self-reported data will be collected by internet based questionnaires (www.checkware.com) before screening, before the rehabilitation program starts, right after the rehabilitation program has ended, and 4, 8 and 12 months after the end of the rehabilitation program. Participants are invited by text messages to access the website and answer the questionnaires.

*Primary outcomes*

- Time from inclusion (i.e. after outpatient screening) until full sustainable return to work (i.e. for at least four weeks without relapse), obtained by national registers.
- Total number of sickness absence days during 12 months of follow-up from inclusion, obtained by national registers.

*Secondary outcomes*

- Readiness, beliefs and motivation for return to work (RRTW) [54].
- Expectations regarding future work participation and sick leave:

*How long do you think you will be on sick leave from today?* (Less than 2 months; 3–6 months; 7–12 months; more than 12 months; I am currently not on sick leave).

*Given your current health situation, do you think you will be receiving full sick-leave benefits six months from now?* (Yes; no).

*Given your current health situation, do you think you will be receiving graded sick leave benefits six months from now?* (Yes, less than 50%; yes, 50% or more).

(The questions were developed during workshops with the research group.)

- Work adjustments and consideration from employer and colleagues:

*Has your employer adjusted your work situation according to your health complaints (*e.g. *changes in work tasks, working hours or facilities)?* (Yes; no; I do not need adjustments).

*To which extent do you feel that your* *employer* *show consideration for your level of functioning?* (Very much; to some extent; not much; I do not need special consideration).

*To which extent do you feel that your* *colleagues* *show consideration for your level of functioning?* (Very much; to some extent; not much; I do not need special consideration)

- Job satisfaction:

*How do you like your work?* (Not at all; not so much; good; very much).

- Return to work self-efficacy (RTWSE-19) [55].
- Health-related quality of life by 15D (15 dimensions) [56].
- Perceived general health:

*How is your health now?* (Poor; not so good; good; very good.)

- Pain intensity and pain sites by a body pain chart and question 3–5 from the Brief Pain Inventory (BPI) [57].
- Physical activity levels measured by three items concerning frequency, intensity and duration of exercise from the third wave of the HUNT study (The HUNT study, Norway. [https://hunt-db.medisin.ntnu.no/hunt-db/#/instrument/130]).
- Fear-avoidance beliefs about work and physical activity [58].
- Psychological flexibility and acceptance [59].
- Psychological flexibility and acceptance related to work [60].
- Perceived work ability:

*How would you rate your current work ability compared with the lifetime best?*

(Scale from 0 to 10, where 10 is best possible work capacity).

- The Hospital Anxiety and Depression Scale (HADS) [61].

#### Economic evaluation

Treatment costs for the workplace intervention and the rehabilitation program will be estimated applying a micro-costing approach. Productivity costs will be calculated from sickness absence data (days) and age and gender specific wage costs from Statistics Norway. Direct costs in terms of participants’ use of health services is recorded by a questionnaire developed and used in a previous study [11]. The questionnaire is answered before the rehabilitation program and 4, 8 and 12 months after the end of the program. It consists of questions concerning the number of:

- days admitted to hospital departments (somatic and psychiatric) or rehabilitation centers
- times receiving medical care (e.g. general practitioner, physiotherapist, chiropractor and occupational care) or alternative treatment (e.g. homeopathy)
- weeks and hours per week of therapeutic work training
- contact-points with the case worker at the The Norwegian Labour and Welfare Service

#### Qualitative studies

Data will be collected through individual interviews with participants, rehabilitations therapists and employers, and through participant observation of the rehabilitation programs and non-participant observation of the workplace intervention. All interviews will be semi-structured and based on an interview guide. Interviews will be audio-recorded and transcribed. Data from observation will be collected through written field notes.

Study A: Therapists will be interviewed individually to investigate how they attend to the return to work process within a rehabilitation program based on ACT. Interviews will take part when the therapists have gained some experience with the program and the intervention. The main topics in the interviews will be what the therapists perceive as the main aim and potential outcomes of the program, and how they attend to the participants’ return to work process during their work. Participant observation of the rehabilitation program will be conducted and will be added to the interviews as data material.

Study B: Approximately 10–15 of the workplace interventions will be followed with non-participant observation. Stakeholders taking part in the workplace intervention (participants, employers and rehabilitation therapists) will be interviewed after the workplace meeting to investigate their experiences. The main topics in the interviews will be the usability and potential outcomes of the current intervention, and how the intervention might be improved. We will aim to include triads with participants, employers and rehabilitation therapists who have attended the same workplace meeting.

Study C: Participants will be interviewed to explore their perceptions of the return to work process four months after the end of the rehabilitation program. Interview topics will be the participants’ experiences of their current work participation and expectations towards the return to work process. Data from interviews will be combined with findings from quantitative data on job satisfaction, return to work self-efficacy, and readiness, expectations, beliefs and motivation for return to work.

### Data analyses

#### Effect evaluation

Effect analyses of primary and secondary outcomes will be performed by intention to treat and per protocol. For the primary outcomes, sustainable return to work will be evaluated with survival analysis and difference in days of sick leave will be evaluated with the Mann Whitney U-test, as sick leave days are not likely to be normally distributed. Effect differences for secondary outcomes will be analysed with linear mixed models or non-parametric methods, as appropriate. Subgroup analyses will be performed for main diagnosis groups.

#### Economic evaluation

We will take the societal perspective and both a cost-effectiveness, cost-utility and cost benefit approach will be applied, and in accordance with the Norwegian guidelines for economic evaluation [62]. In the cost-effectiveness analysis, sickness absence days will be used as outcome measure, and to avoid double counting productivity costs will be excluded. In the cost-utility analysis, the outcome measure is Quality Adjusted Life Years [63] based on the 15D instrument [56], and cost-utility estimates will be provided both with and without the inclusion of productivity costs. The cost-benefit analysis will compare costs and benefits taking increased treatment costs and potential societal savings from reduced sickness absenteeism into account. Analyses of cost-effectiveness and cost-utility will be performed by calculating the ratio of incremental costs divided by incremental effect. Univariate sensitivity analyses and bootstrapping procedures will be applied to estimate uncertainty surrounding the incremental ratios.

#### Qualitative studies

Analysis of interviews will be inspired by interpretive phenomenological analysis (IPA) [64], while analysis of the observational studies will be inspired by thematic analysis [65].

## Ethics

The Regional Committee for Medical and Health Research Ethics in Central Norway has approved the protocol (No.: 2014/2279), and the trial is registered in clinicaltrials.gov (NCT02541890). All participants who are included in qualitative observation and interview studies will sign an informed consent before they take part in the study.

## Discussion

As described in the Background, designs suitable to encompass complex and novel interventions provided to a heterogeneous patient group are wanting. Researchers have emphasized the need for good quality evaluations of initiatives intended to enhance return to work [66]. The study described in this protocol article will combine an effect evaluation, economic evaluations, and qualitative and mixed methods study in a comprehensive approach.

Although different countries have different arrangements for follow-up and benefits during sickness absence, the results from this study could be useful for contexts similar to the Norwegian. The rehabilitation program provided in this study takes place in an in-patient rehabilitation center setting, but is not dependent on this setting to be replicated. Any results regarding effect on return to work or health outcomes should thus be generalizable to other contexts. Since the intervention takes place at the participant’s workplace, it would be useful for other contexts, and the intervention in itself is applicable in most workplace settings. The design of this study will make it possible to assess effects of the program, both regarding work participation and economic benefit. In addition to providing results in its own right, qualitative studies nested in randomized controlled trials are recommended as a way to enhance the significance of the results [67–69]. The qualitative results could also be helpful in modifying and applying the different parts of the rehabilitation program and the workplace meeting into other contexts.

The heterogeneous patient group as well as the complexity of the rehabilitation program and the workplace intervention, are all aspects which make a good and robust study design very important. Adding the patients’ perspectives in qualitative studies might give valuable insight for the development of further studies. The exploration of the different stakeholders’ perspectives and experiences with the same workplace intervention could provide new and interesting knowledge on the suitability of such interventions, as well as any barriers which are not accounted for in the development of the intervention. Input from patients and employers will be very important to make necessary improvements.

Although the researchers are blinded during the analyses, this study’s randomized controlled design does not allow for any blinding of the participants or rehabilitation therapists. The rehabilitation programs in this study are designed according to the Norwegian systems and culture - regarding benefits, work, health, and policies. The results from the study are thus not necessarily generalizable to countries with different socio-economic and socio-cultural contexts than Norway. To implement similar rehabilitation programs in other countries necessary modifications should be considered.

### Impact of results

Investigating the stakeholders’ experiences and evaluations are necessary supplements to studies investigating the quantitative effects from an intervention. The combination of quantitative and qualitative data will provide knowledge on important individual and contextual causal and predictive factors during the return to work process. Professionals providing health and work services will benefit from the results in how to best support sick-listed persons to return to work. The results on effect and cost-effectiveness generated in this study will give policy makers a knowledge base for making decisions for future policies in this field.
